# Deep eutectic solvent-Ferrofluid based single-step magnetic-assisted liquid-liquid microextraction for Pyrethroid residues determination in vegetable oils by GC–MS/MS

**DOI:** 10.1016/j.fochx.2026.103576

**Published:** 2026-01-24

**Authors:** Jingjing Yu, Yuxin Liu, Cong Wang, Mantang Chen, Cong Nie, Wei Liu

**Affiliations:** a Zhengzhou Tobacco Research Institute of CNTC, Zhengzhou 450001, PR China; b College of Food Science and Technology, Henan University of Technology, Lianhua Street, Zhengzhou 450001, PR China

**Keywords:** Ferrofluids, Deep eutectic solvent, Pyrethroids, Vegetable oils, GC–MS/MS

## Abstract

A deep eutectic solvents-based ferrofluids (DES-FFs) material was prepared by combining small-sized Fe_3_O_4_ magnetic cores with a ternary choline chloride/sesamol/coumarin (1:3:1) DESs. The material was used for single-step magnetic-assisted liquid-liquid microextraction (MALLME) and determination of 15 pyrethroids (PYs) in vegetable oils by GC–MS/MS. The small-sized Fe_3_O_4_ material improved colloidal stability of DES-FFs and enabled rapid phase separation (<10 s). The DES-FFs might leverage enhanced π-π stacking interactions between sesamol/coumarin and PYs, and optimal hydrophilicity-polarity matching, which collectively contributed to the high extraction efficiency for PYs. Comprehensive characterization (HRTEM, XRD, FTIR, TGA, etc.) validated the material's structural integrity, thermal stability and magnetic property. The developed method demonstrated high sensitivity (LODs: 0.91–3.03 ng/mL; LOQs: 3.02–10.09 ng/mL), significantly reduced matrix effect, and good precision (RSD ≤6.92%) for 15 PYs in vegetable oils, with recoveries ranging from 79.88% to 107.90%. Determination of 15 PYs was successfully achieved in both refined and crude vegetable oils.

## Highlights

• A DES-FFs material was prepared by combining Fe_3_O_4_ magnetic cores with a ternary choline chloride/sesamol/coumarin-DES.
• The material was used for single-step MALLME and determination of PYs in vegetable oils by GC–MS/MS.
• High extraction efficiency of PYs using DES-FFs might be due to the enhanced π-π stacking interaction.
• Small-sized magnetic nanoparticles improved the colloidal stability of DES-FFs and enabled rapid phase separation (< 10 s).

## 1 Introduction

Vegetable oils, as major lipid source in the global food supply chain, their safety and quality are directly related to human health and international trade. Pesticides are widely used to control moulds, insects, or weeds during various cultivation stages to increase crop yield in modern intensive agriculture (Alabi et al., 2024; Anagnostopoulos et al., 2025; Aralimarad et al., 2025). The application of pesticides in oilseed crop cultivation has been identified as a primary factor contributing to the increasing presence of pesticide residues in vegetable oils. The high binding affinity and structural stability of fat-soluble pesticides dissolved in vegetable oils lead to their incomplete removal during oil refining processes. This leads to pesticide residues in vegetable oils with a concentration range of μg/kg to mg/kg, which poses significant food safety risks (Laoretani & Fischer, 2023; Liu et al., 2013). As demonstrated by China's 2021 monitoring data, pesticide residues were detected in 52.2% of various vegetable oils, including peanut oil, olive oil, soybean oil, rapeseed oil and sesame oil (Hou et al., 2021). Consequently, meticulous monitoring of pesticide residue levels in both crude and refined vegetable oils is imperative.

Pyrethroids (PYs) are synthetic analogs of pyrethrins and constitute an important class of synthetic insecticides characterized by high efficiency, broad-spectrum activity, low toxicity and biodegradability. Significant efforts to develop PYs insecticides were undertaken in the late 1960s and particularly throughout the 1970s. A major milestone was achieved in 1973 with the successful development of the first sunlight-stabilized pyrethroid, permethrin, which overcame the shortcomings of instability in light and air inherent to natural pyrethrins. Today, over 70 types of PYs, including permethrin, cypermethrin, deltamethrin, fenvalerate, and bifenthrin, are widely used in agriculture on various fruits and vegetables. Consequently, PYs have also been detected in vegetable oils (Esteve-Turrillas et al., 2005; Farajzadeh et al., 2014; Yu et al., 2017). Maximum residue limits (MRLs) for PYs have been established for various types of vegetable oil in certain jurisdictions, covering both crude and refined oils. For instance, the Chinese National Standard (Standardization Administration of China, 2021) sets PYs MRLs ranging from 0.02 to 0.5 mg/kg in cottonseed oil, rapeseed oil, soybean oil, sunflower oil and olive oil, while MRLs for specific crude oils range from 0.1 to 3.0 mg/kg. The European Union has established MRLs for PYs in vegetable oil raw materials (e.g., cottonseeds, rapeseeds, soybeans, sunflower seeds, and olives) ranging from 0.01 to 0.7 mg/kg (European Commission, 2023), though not specifically for crude and refined oils.

Although strict MRLs of PYs have been established, the analytical challenges posed by oil matrices severely limit detection efficacy. The “matrix effect”, caused by the high triglycerides content in vegetable oils, significantly impedes the accurate detection of trace PYs and other pesticide residues (Ma et al., 2016). This problem is generally addressed by means of complex sample pretreatment methods prior to instrument analysis. Conventional methodologies involve a clean-up step using solid phase extraction (SPE) / liquid-liquid microextraction (LLE) (Deme et al., 2013; Esteve-Turrillas et al., 2005; Farajzadeh et al., 2014; Muccio et al., 1999; Patel et al., 2005; Zehringer & Herrmann, 2001; Zrostlikova et al., 2002), or gel permeation chromatography (GPC) (Patel et al., 2005; Zrostlikova et al., 2002), followed by extracting with various organic solvents. For example, Esteve-Turrillas and co-workers (Esteve-Turrillas et al., 2005) developed a method for determining PYs in vegetable oils. Oil samples were partitioned with acetonitrile-hexane (1:1, *v*/*v*) and then eluted through a combined column packed with deactivated basic alumina and C_18_. Eleven PYs were determined from olive, sunflower, corn and soybean oils using a gas chromatography–mass spectrometry (GC–MS) method. Farajzadeh and co-workers (Farajzadeh et al., 2014) developed a method to extract 5 PYs from vegetable oils which was followed by a Gas chromatography - Flame ionization detector (GC-FID) and GC–MS determination. In their studies, oil samples were initially partitioned with a dimethylformamide (DMF)-hexane mixture. The DMF was removed and then directly used as a disperser solvent in the following dispersive liquid-liquid microextraction (DLLME) procedure, in which μL-level of 1,1,2-trichloroethane was used as an extraction solvent. Patel et al. (2005) used the GPC method in conjunction with the GC–MS/MS analysis to detect 19 organochlorine pesticides in fats and oils. But the liner of the GC–MS/MS instrument needed to be replaced daily. GPC is considered as a relatively effective method of oil removal. However, GPC equipment is expensive, and costly to maintain. And it is difficult to completely remove all interfering substances from oils using only GPC. Moreover, the other multi-step pretreatment methods reported in the literature are quite complex and time-consuming, which seriously restricts experimental efficiency. To enhance sample pretreatment efficiency for the detection of PYs and other pesticides in oil matrices, various novel and simplified methods have been developed (Deme et al., 2014; Li et al., 2014; Sani et al., 2023; Yu et al., 2017). Yu et al. (2017) reported an efficient and convenient clean-up method at low temperature. The determination of 8 PYs in vegetable oils was achieved by combining magnetic nanoparticle-based SPE with a high performance liquid chromatography - diode array detector (HPLC-DAD) method. The oil froze, leaving the organic solvent in the liquid supernatant at the low temperature stage, enabling easy separation of the oil. However, the low-temperature treatment process is still cumbersome.

Recently, deep eutectic solvents (DESs) are widely used as extractants for the extraction of trace compounds from complex matrices with superior extraction performance and efficiency (Boateng, 2022; He et al., 2024; Wang & Liu, 2025). DESs are usually environmentally friendly, inexpensive, and easy to prepare. However, separating DESs after liquid-liquid extraction is difficult because they are in liquid form. Centrifugation and pipetting are usually required. Ferrofluids (FFs) based on DESs are a new type of magnetic nanofluids. In this system, DESs are coated on the surface of magnetic nanoparticles. FFs based on DESs can use magnetic fields to facilitate separation. This approach has been demonstrated to provide a simpler and more effective method compared with conventional DESs-based microextraction techniques (Cao et al., 2024; Cheng et al., 2025; Dil et al., 2019; Jouyban et al., 2020; Liu et al., 2025; Ortizo et al., 2024). Cao et al. (2024) reported that Fe_3_O_4_ nanoparticles were coated with water-based choline chloride (ChCl) DESs to prepare a magnetic FF. The material was successfully used to enrich and detect two types of plant growth regulators (1-naphthylacetic acid and 2-naphthylacetic acid) in edible oil, coupled with the HPLC-ultraviolet detection (UV) technique. Dil et al. (2019) prepared FFs containing magnetic nanoparticles and oleic acid, combined with hydrophobic DESs. It was used to extract and determine methoxyamine acid (MMA) in urine samples with a HPLC-UV method by the vortex-assisted liquid-liquid microextraction. After the extraction process, the FF material was separated by an external magnetic field. It is evident that the magnetic-assisted liquid-liquid microextraction (MALLME) using FFs based on DESs (DES-FFs) provides a simple and high-efficient pretreatment platform for the enrichment of trace compounds in complicated sample matrices. It is important to note that a significant number of studies on DES-FFs had employed the approach of coating the common sized-magnetic nanoparticles with various surfactants, such as oleic acid (Dil et al., 2019; Liu et al., 2025) and Aliquat 336 (Jouyban et al., 2020), prior to preparing DES-based FFs. This approach was used to address the instability of DES-FFs, which was attributed to the aggregation and precipitation of magnetic nanoparticles (Cheng et al., 2025). It introduced an additional step to the material synthesis process, which was both time-consuming and troublesome.

In this study, a novel DES-FFs-based MALLME method was developed for the first time to determine 15 PYs in vegetable oils in conjunction with the GC–MS/MS technique. The synthesis of small-sized Fe_3_O_4_ nanoparticles was undertaken for the innovative preparation of DES-FFs without the addition of any surfactant. The stability of the DES-FFs was found to be enhanced in comparison to those prepared for common sized-Fe_3_O_4_ nanoparticles. In order to balance the polarity of PYs, oils and elute solvents, and thereby achieve high extraction efficiency of PYs and suppress the matrix effects of oil samples, a ChCl/sesamol/coumarin (COU) DES (the molar ratio of three components was 1:3:1) was designed and synthesized for the preparation of DES-FFs. Consequently, a single-step MALLME with DES-FFs materials composed of ChCl/sesamol/COU DES and small-sized Fe_3_O_4_ nanoparticles was developed in this study. The present study focused on the optimization of the MALLME technique and the evaluation of the performance of the developed method. The practical application of the developed method was examined on refined and crude vegetable oils. The utilization of ChCl/sesamol/COU-based DES-FFs, incorporating small-sized Fe_3_O_4_ nanoparticles, has been demonstrated to facilitate a simpler, faster single-step MALLME platform for the analysis of targets in fats and oils samples.

## 2 Materials and methods

### 2.1 Chemicals and reagents

Various refined and crude vegetable oils were purchased from local markets in Zhengzhou (China), including cottonseed oil, rapeseed oil, sunflower oil, olive oil and soybean oil. Standard substances of 15 PYs including tefluthrin, bifenthrin, fenpropathrin, lambda-cyhalothrin, trans-permethrin, cis-permethrin, cyfluthrin, cypermethrin, flucythrinate, beta-cypermethrin, etofenprox, fenvalerate, fluvalinate, esfenvalerate, and deltamethrin (all≥99.9%) were obtained from Sigma-Aldrich (USA). Anhydrous betaine (BET) and ChCl (all≥98%) were purchased from Sigma-Aldrich (USA) as well. Ammonium ferrous sulfate hexahydrate (Fe(NH_4_)_2_·(SO_4_)_2_·6H_2_O) (≥98%), ferric ammonium sulfate dodecahydrate (NH_4_Fe(SO_4_)_2_·12H_2_O) (≥99.5%), FeCl_3_·6H_2_O (ACS), ethylene glycol (≥99.5%), anhydrous sodium acetate (≥99%), Triton X-100 (TX-100, UltraBio™), sesamol (98%), coumarin (COU, ≥99.5%), heptachlor epoxide B (≥99.9%), H_2_SO_4_ (95–97%) and NaOH (≥98%) were purchased from Aladdin Biochemistry & Technology Co., Ltd. (China). Ethanol, acetonitrile, methanol, *n*-hexane, dichloromethane, and ethyl acetate were analytical reagents and purchased from Dikma Technologies Inc. (China). The standard solutions of PYs were prepared with *n*-hexane.

### 2.2 Synthesis of Fe_3_O_4_ nanoparticles

Two kinds of Fe_3_O_4_ nanoparticles with different sizes were synthesized in this study. The small-sized Fe_3_O_4_ nanoparticles were synthesized according to the method reported in the reference (Mandal et al., 2005). An iron source solution was prepared by dissolving 0.128 mol NH_4_Fe(SO_4_)_2_·12H_2_O and 0.064 mol Fe(NH_4_)_2_·(SO_4_)_2_·6H_2_O in 100 mL of 0.40 mol/L H_2_SO_4_ solution. A certain amount of TX-100 solution was added to 250 mL of 1.0 mol/L NaOH solution, and the final concentration of TX-100 was 0.01 mol/L. Then, the mixture was heated to 80 °C. During this process, 25 mL of the iron source solution was added drop by drop to the mixture while it was being stirred vigorously. Stirring continued for 30 min. The temperature of the entire process was maintained at approximately 80 °C. The mixture was then left to cool to room temperature, after which the particles in the solution were separated using a magnet. The small-sized Fe_3_O_4_ nanoparticles obtained were washed for 5 times with 40 mL water each time and then 3 times with 20 mL anhydrous ethanol each time, and then dried for 12 h in a vacuum oven at 50 °C. The small-sized Fe_3_O_4_ nanoparticles were named as S-Fe_3_O_4_.

For comparison, common Fe_3_O_4_ nanoparticles were synthesized using the method described in the reference (Yu et al., 2020). Briefly, 4.0 g of FeCl_3_·6H_2_O was dissolved in 120 mL of ethylene glycol under mechanical agitation at 50 °C. After complete dissolution, 8.0 g of anhydrous sodium acetate was added and the mixture was agitated for 60 min. The mixture was then transferred to a hydrothermal reactor and heated to 200 °C for 6 h. Purification was conducted by separating the nanoparticles using a magnet, followed by washing them sequentially with deionized water and anhydrous ethanol, repeating each step 3–5 times. The resulting Fe_3_O_4_ nanoparticles were vacuum dried at 50 °C for 12 h, and named as C-Fe_3_O_4_.

### 2.3 Synthesis of DESs

Four types of DESs were synthesized using appropriate molar ratios of hydrogen bond acceptor (HBA) and hydrogen bond donor (HBD). The HBA and HBD were mixed and stirred continuously at 80 °C or 90 °C about 2 h to form a clear, homogeneous liquid. Table 1 provided detailed information of the synthesized DESs.

**Table 1:** The composition information of DESs.

DESs abbreviation	Composition	Molar ratio
ChCl/sesamol	Choline chloride, Sesamol	1:2
ChCl/sesamol	Choline chloride, Sesamol	1:3
BET/sesamol	Betaine, Sesamol	1:3
ChCl/sesamol/COU	Choline chloride, Sesamol, Coumarin	1:3:1

### 2.4 Preparation of DES-FFs

**Fig. 1:**
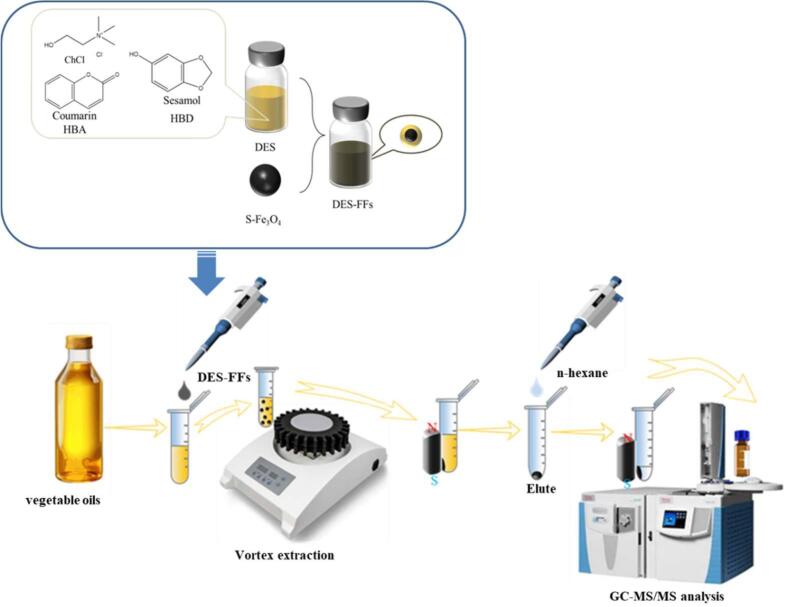
Flow chart of the preparation of DES-FFs and the process of MALLME-GC–MS/MS method based on DES-FFs.

To prepare DES-FFs, 50 mg of small-sized Fe_3_O_4_ nanoparticles (S-Fe_3_O_4_) were dispersed in 1 mL of DESs. The mixture was sonicated at 40 KHz for about 20 min (KQ-700DE Model CNC Ultrasonic Cleaner, Kunshan Ultrasonic Instrument Co., Ltd., China) to disperse any nanoparticle clusters, after which the DES-FFs material was obtained. Fig. 1 demonstrated a schematic graphic showing the preparation of DES-FFs. For comparison, common Fe_3_O_4_ nanoparticles (C-Fe_3_O_4_) were also dispersed in DESs to prepare DES-FFs.

### 2.5 Materials characterization

Various modern instrumental testing techniques were employed to confirm the composition and properties of the prepared materials. The thermal stability and transition properties of the prepared DESs were assessed using a thermogravimetric analyzer (TGA, Model 550, Waters, USA) and differential scanning calorimetry (DSC, Discovery, TA, USA). TGA measurement was conducted under an N_2_ atmosphere at temperatures ranging from 60 °C to 350 °C, with a heating rate of 10 °C/min. The DSC test was conducted under an N_2_ atmosphere ranging from −90 °C to 25 °C at a constant rate of 10 °C/min. Fourier transform infrared spectroscopy (FTIR, Bruker Tensor 27, Germany) was used to investigate the functional groups and to ensure that the DESs components had been formed correctly. The surface charge of the Fe_3_O_4_ nanoparticles was tested using zeta potential analysis (Malvern Mastersizer Nano ZS ZEN 3600, UK). A high-resolution transmission electron microscope (HRTEM, JEOL JEM-2100F, Japan) and a powder X-ray diffraction (XRD, Bruker D8 Venture, Germany) were used to investigate the topography and structure of the nanoparticles. The magnetic properties of DES-FFs and Fe_3_O_4_ nanoparticles were evaluated using a PPMS-9 integrated physical property measurement system (Quantum Design, USA).

### 2.6 MALLME with DES-FFs material

5 mL of the vegetable oil sample was added to a 10 mL centrifuge tube. Then, a certain amount of internal standard solution (heptachlor epoxide B) was added to achieve a final concentration of 0.1 mg/L. Subsequently, 100 μL of DES-FFs was added dropwise to the oil sample. Following vortex extraction at 2000 rmp for 15 min using a Heidolph Tube Shaker Muti Reax (Germany), the DES-FFs phase was separated using an external magnetic field. The magnet was placed on the outer wall of the centrifuge tube to attract the DES-FF phase, after which the oil solution was poured out. Then, 1 mL n-hexane was added as an eluent solution to extract the PYs from the DES-FFs by vortexing for 15 min. After separating the phases using an external magnetic field, the n-hexane phase was collected and filtered through a 0.22 μm organic phase nylon syringe filter, and then analyzed using a GC–MS/MS method. Fig. 1 showed the flow chart of the preparation of DES-FFs and the procedure of the DES-FFs based MALLME-GC–MS/MS method. A simulated oil sample was prepared by spiking the cottonseed oil sample with 200 ng/mL PYs for optimizing MALLME conditions.

### 2.7 GC–MS/MS conditions

PYs were determined using a GC–MS/MS system comprising a TRACE 1610 Gas Chromatograph and TSQ 9610 Triple Quadrupole Mass Spectrometer (both from Thermo Fisher Scientific, USA). The column was an Agilent DB-5MS column (60 m × 250 μm × 0.25 μm). The temperature program was as follows: an initial hold at 40 °C for 1 min, then ramping to 250 °C at 30 °C/min, to 280 °C at 6 °C/min, to 300 °C at 10 °C/min, and finally holding at 300 °C for 5 min. High-purity helium (≥99.999%) was used as the carrier gas. The column flow was 1.0 mL/min and the purge flow was 5.0 mL/min. The inlet temperature was maintained at 280 °C, and 1 μL of the sample was injected in splitless mode.

Mass spectrometric detection was performed using an electron ionization (EI) source (70 eV, 290 °C), with the transfer line set to 280 °C. The solvent delay time was 7.0 min. The MS/MS collision gas was argon (Ar). Data were acquired in total ion chromatogram (TIC) and selective reaction monitoring (SRM) modes. The detailed SRM parameters for the 15 PYs and the internal standard (heptachlor epoxide B) were provided in Table 2.

**Table 2:** SRM parameters of compounds.

Compounds	Retention time/min	Precursor and Products ions /(*m*/*z*)	Dwell time/s	Collision energy/V
tefluthrin	11.709	177.0/127.1^a^	0.2	14
		177.0/137.1^b^	0.2	14
heptachlor-epoxide B	13.923	352.8/262.9^a^	0.2	12
		352.8/128.0^b^	0.2	12
bifenthrin	17.507	181.1/165.1^a^	0.2	24
		181.1/166.2^b^	0.2	10
fenpropathrin	17.928	97.1/41.1^a^	0.2	16
		97.1/55.1^b^	0.2	8
lambda-cyhalothrin	19.389	197.0/141.1^a^	0.2	10
		197.0/161.1^b^	0.2	6
trans-permethrin	21.748	183.1/165.1^a^	0.2	10
		183.1/168.1^b^	0.2	10
cis-permethrin	22.104	183.1/153.1^a^	0.2	12
		183.1/168.1^b^	0.2	10
cyfluthrin	24.048	163.0/91.1^a^	0.2	12
		163.0/127.1^b^	0.2	6
cypermethrin	24.619	165.0/91.1^a^	0.2	12
		165.0/127.1^b^	0.2	6
flucythrinate	25.259	199.1/107.1^a^	0.2	20
		199.1/157.1^b^	0.2	8
beta-cypermethrin	25.414	165.0/91.1^a^	0.2	12
		165.0/127.1^b^	0.2	6
etofenprox	26.060	163.1/107.1^a^	0.2	16
		163.1/135.1^b^	0.2	8
fenvalerate	28.762	125.0/89.1^a^	0.2	18
		125.0/99.1^b^	0.2	20
fluvalinate	29.165	250.1/55.1a	0.2	14
		250.1/200.1^b^	0.2	16
esfenvalerate	29.580	125.0/89.1^a^	0.2	18
		125.0/99.1^b^	0.2	18
deltamethrin	31.604	252.9/93.1^a^	0.2	16
		252.9/174.0^b^	0.2	6

^a^: Quantitative ion; ^b^: Qualitative ion.

## 3 Results and discussion

### 3.1 Synthesis and characterization of the materials

As shown in Fig. 1, HBD and HBA formed DESs through hydrogen bonding, maintaining a uniform liquid state at room temperature. The DESs were coated onto Fe_3_O_4_ nanoparticles to yield the DES-FFs material. Generally, ChCl-based DESs are highly hydrophilic. The abundant hydrophilic groups (e.g., hydroxyl groups) on the synthesized Fe_3_O_4_ nanoparticles facilitated the attachment of the ChCl-based DESs to the magnetic nanoparticles, thus producing the DES-FFs.

**Fig. 2:**
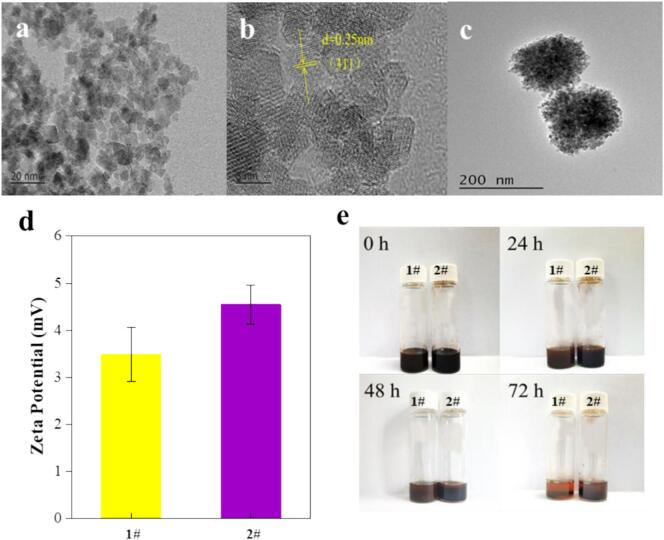
(a, b) HTEM images of small-sized Fe_3_O_4_ nanoparticles (S-Fe_3_O_4_); (c) HTEM image of common Fe_3_O_4_ nanoparticles (C-Fe_3_O_4_); (d) Zeta potential values of DES-FFs prepared with C-Fe_3_O_4_ (1#) and S-Fe_3_O_4_ (2#); (e) Stability of DES-FFs prepared with C-Fe_3_O_4_ (1#) and S-Fe_3_O_4_ (2#) after different standing times.

To improve the stability of the DES-FFs material, small-sized Fe_3_O_4_ nanoparticles (S-Fe_3_O_4_) were synthesized by the hydro-micellar method, while common Fe_3_O_4_ nanoparticles (C-Fe_3_O_4_) were synthesized by the hydrothermal method for comparison. As shown in the HRTEM images (Fig. 2a), S-Fe_3_O_4_ had a particle size of 7.11 ± 1.39 nm, providing better dispersion in DESs. The lattice stripe spacing of 0.25 nm corresponded to the (311) crystal plane of S-Fe_3_O_4_ (Fig. 2b), with the crystal structure was confirmed by XRD (**Fig. S1**). C-Fe_3_O_4_ exhibited a larger particle size of 158.89 ± 6.43 nm (Fig. 2c). Zeta potential analysis of the two types of DES-FFs materials (Fig. 2d) showed significantly higher values for that based on S-Fe_3_O_4_ (4.55 ± 0.42 mV) than that for C-Fe_3_O_4_ (3.49 ± 0.57 mV), indicating enhanced stability of S-Fe_3_O_4_-based DES-FFs. Storage stability tests (Fig. 2e) demonstrated that S-Fe_3_O_4_ remained well-dispersed in DESs after 48 h, whereas C-Fe_3_O_4_-based DES-FFs underwent delamination within 24 h due to nanoparticle aggregation. Thus, the utilization of S-Fe_3_O_4_ in the preparation of the DES-FFs enhanced their stability, ensuring a homogeneous fluid state and enabling rapid phase separation in less than 10 s. In a conventional approach outlined in the literature (Dil et al., 2019; Jouyban et al., 2020; Liu et al., 2025), common Fe_3_O_4_ nanoparticles were coated with various surfactants to enhance the stability of DES-FFs. By contrast, preparing DES-FFs using S-Fe_3_O_4_ in this study offered distinct advantages, as it demonstrated excellent stability without the need to introduce surfactant. Thus, small-sized Fe_3_O_4_ nanoparticles were selected for the preparation of DES-FFs.

**Fig. 3:**
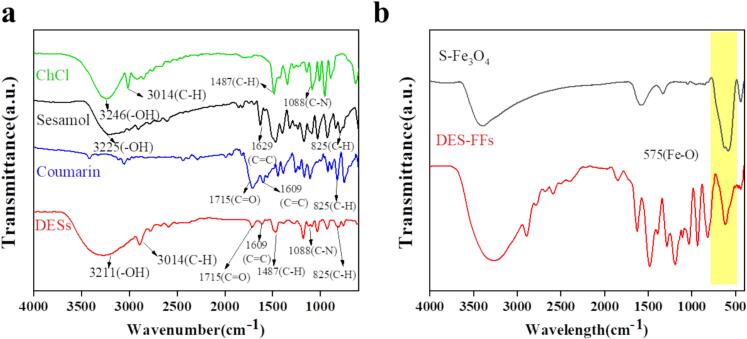
(a) FTIR spectra of DESs, coumarin, sesamol, ChCl; (b) FTIR spectra of small sized Fe_3_O_4_ (S-Fe_3_O_4_) and DES-FFs.

As shown in Fig. 3a, the FTIR spectra of both DESs and ChCl exhibited stretching vibration bands of -CH_3_ at 3014 cm^−1^ and C—H bending vibration peaks at 1487 cm^−1^. The aromatic ring C=C stretching vibration (shared by sesamol and COU) and C=O stretching vibration (characteristic of COU) appeared in the spectrum of DESs. Corresponding signals were also observed for aromatic C—H bending in the fingerprint region, indicating that DESs synthesis preserved the molecular structures of HBA and HBD. Crucially, the -OH absorption peak of the DESs at 3211 cm^−1^ showed a significant red shift compared to that of sesamol (3225 cm^−1^) and ChCl (3246 cm^−1^), with peak broadening and intensified absorption suggesting hydrogen bond formation. Fig. 3b showed FTIR data for DES-FFs prepared with small-sized Fe_3_O_4_ nanoparticles. The characteristic Fe—O stretching vibration at 580 cm^−1^ confirmed the intact Fe_3_O_4_ structure in the DES-FFs. To further verify the formation of the ChCl/sesamol/COU-based DESs, the thermal stability and phase behavior were analyzed by TGA (**Fig. S2**) and DSC (**Fig. S3**). The TGA curves indicated that the DESs exhibited lower thermal stability than their individual components, consistent with literature reports (Farajzadeh et al., 2014). DSC analysis revealed only a glass transition at −67.06 °C, which was significantly lower than the melting points of ChCl (302 °C), sesamol (62–65 °C), and COU (68–70 °C). These results collectively confirmed the successful formation of the DESs.

**Fig. 4:**
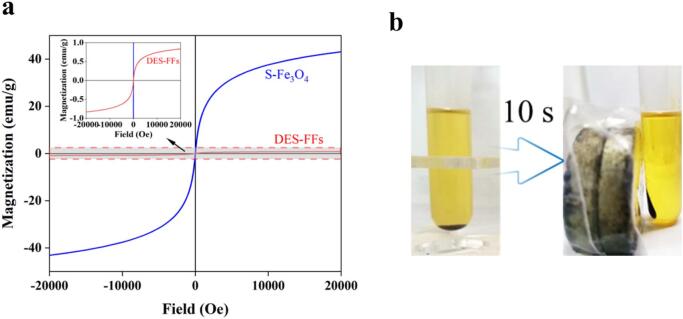
(a) Hysteresis loops of small sized Fe_3_O_4_ (S-Fe_3_O_4_) and DES-FFs; the inset shows the enlarged hysteresis loop of DES-FFs. (b) The fluid state of DES-FFs after magnetic separation from the oil phase.

Fig. 4a showed the hysteresis loops of small-sized Fe_3_O_4_ and the DES-FFs based on it, indicating superparamagnetic behavior in both materials. The saturation magnetization (*Ms*) values were 43.19 *emu/g* for Fe_3_O_4_ and 0.83 *emu/g* for DES-FFs at 300 K. This reduction was attributed to the DESs coating on the magnetic nanoparticles. Although the magnetization decreased significantly, the DES-FFs retained adequate magnetism for rapid collection (<10 s) under an external magnetic field, eliminating the need for centrifugal separation. Fig. 4b showed the homogeneous fluid state of the DES-FFs after magnetic separation from the oil phase.

### 3.2 Enrichment of PYs with DES-FFs based MALLME

**Fig. 5:**
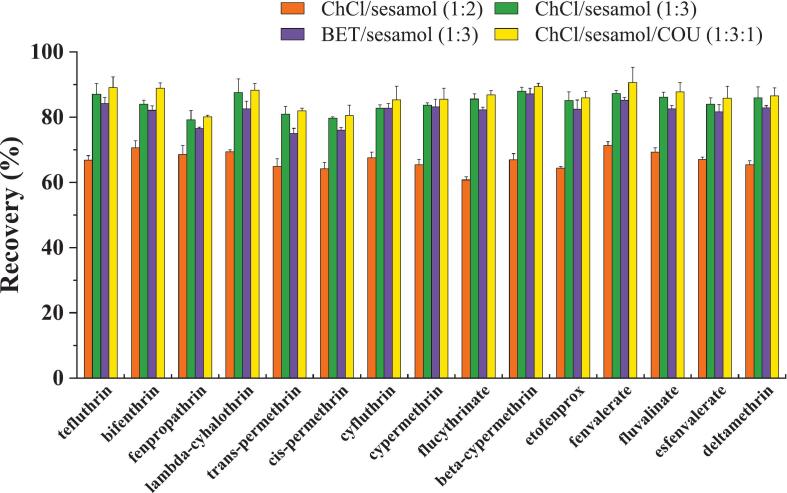
Effect of DES-FFs with different DESs on the extraction recovery of 15 PYs. It shows the results of three parallel measurements. Error bars represents the standard deviation.

**Fig. 6:**
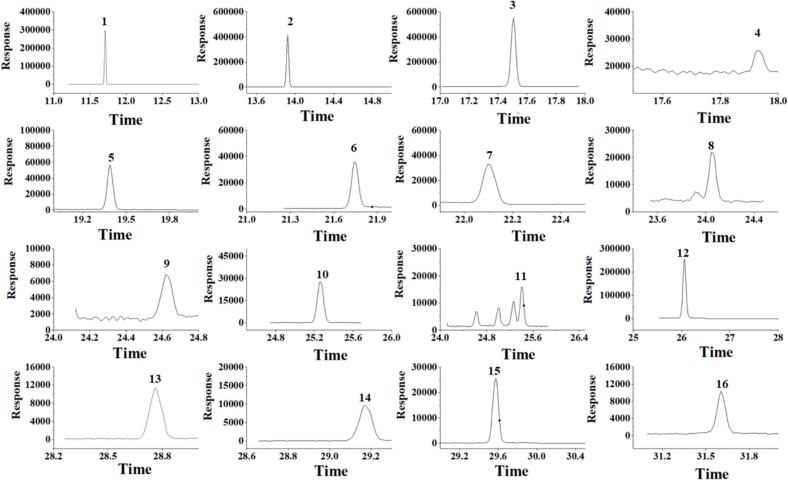
SRM chromatograms of 15 PYs and internal standard (IS) in spiked cottonseed oil sample after MALLME procedure. 1-tefluthrin; 2- heptachlor-epoxide B (IS); 3-bifenthrin; 4-fenpropathrin; 5-lambda-cyhalothrin; 6-trans-permethrin; 7-cis-permethrin; 8-cyfluthrin; 9-cypermethrin; 10-flucythrinate; 11-beta-cypermethrin; 12-etofenprox; 13-fenvalerate; 14-fluvalinate; 15-esfenvalerate; 16-deltamethrin.

As demonstrated in earlier studies (Khezeli et al., 2015; Liu et al., 2019; Liu et al., 2025), the presence of benzene rings in DESs has been shown to enhance the extraction and enrichment efficiency for target compounds of a similar structure. It is hypothesized that the most probable extraction mechanism is thought π-π stacking interactions between the DESs and the target compounds (Khezeli et al., 2015; Liu et al., 2019; Liu et al., 2025). The chemical structure of PYs is characterized by the presence of benzene rings, suggesting potential π-π stacking interactions with benzene-containing DESs monomers. Additionally, the polarity difference between DESs and vegetable oil is critical for MALLME, as it enables the extraction of PYs while preventing the dissolution of DESs in the oil. The polarity of DESs also ensures an effective nanoparticle coating. The successful implementation of MALLME requires all three conditions. Based on these principles, four benzene-containing DESs were synthesized (Table 1). The corresponding FFs served as magnetic adsorbents for extracting 15 PYs from spiked cottonseed oil. The extraction efficiencies (Fig. 5) revealed that the ChCl/sesamol/COU (1:3:1)-based DES-FFs outperformed the others, achieving the highest recoveries of PYs. Well-defined peaks in SRM chromatograms (Fig. 6) confirmed the suitability for quantitative analysis. This superior performance resulted from the dual benzene-ring HBDs (sesamol and COU) at high molar ratios (ChCl/sesamol/COU, 1:3:1), which might enhance the π-π interactions. Furthermore, the ternary DESs combined with ChCl, sesamol and COU at a molar ratio of 1:3:1 was found to possess suitable hydrophilicity and polarity, as well as adequate viscosity for MALLME of PYs from vegetable oil matrices.

### 3.3 Optimization of MALLME conditions

To achieve the best extraction efficiency, the MALLME conditions were optimized, including the DES-FFs dosage, extraction time, elution solvent type and elution time. Extraction efficiency was characterized by the recoveries of 15 PYs in spiked cottonseed oil samples, where a higher recovery indicated higher the extraction efficiency.

**Fig. 7:**
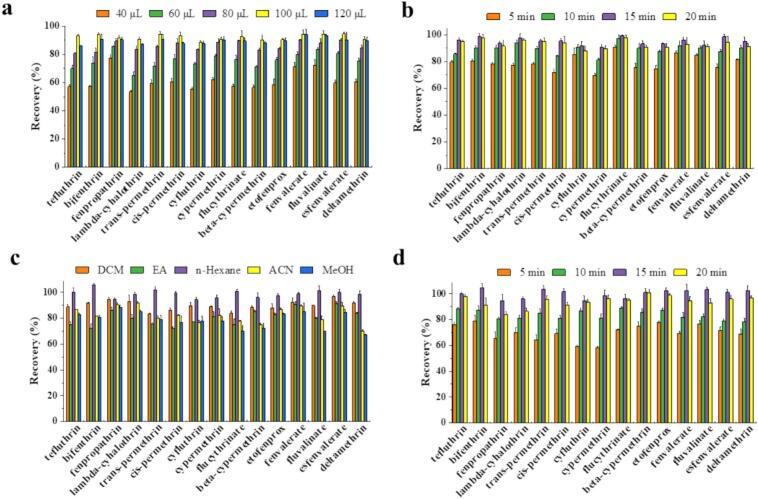
DES-FFs dosage (a), extraction time (b), elution solvent (c) and elution time (d) on the extraction recovery of 15 PYs (*n* = 3). Elution solvents contain dichloromethane (DCM), ethyl acetate (EA), n-hexane, acetonitrile (ACN) and methanol (MeOH). Experimental conditions: 5 mL of oil sample; PYs spiked concentration 200 ng/mL. It shows the results of three parallel measurements. Error bars represents the standard deviation.

As shown in Fig. 7a, the recoveries of PYs increased with the DES-FFs dosage. Maximum recoveries were achieved for all 15 PYs at 100 μL of DES-FFs. Increasing the dosage to 120 μL resulted in plateau or slightly reduced recoveries, indicating 100 μL as the optimal dosage. Fig. 7b demonstrated the recoveries of PYs during 5–15 min of vortex extraction. The recoveries of all PYs reached peak values at 15 min, with no significant increase at 20 min, confirming that extraction equilibrium was attained within 15 min. The elution solvent significantly affected the extraction efficiency (Fig. 7c). When acetonitrile and methanol were used as elution solvents, their strong polarity caused the DESs coating (ChCl/sesamol/COU, 1:3:1) to dissolve during elution. However, when the eluate was injected into GC–MS/MS, it caused severe signal suppression. Dichloromethane as an elution solvent similarly dissolved the DESs coating. Ethyl acetate as an elution solvent caused the DES-FFs material to decompose, which was incompatible with MALLME. Consequently, these solvents yielded lower extraction efficiency, as shown in Fig. 7c. In contrast, n-hexane provided the highest recoveries of PYs while preserving the structural integrity of the DES-FFs, ensuring MALLME compatibility. Thus, n-hexane was selected as elution solvent in this study. Elution time optimization (Fig. 7d) showed that the recoveries of PYs increased with vortex time (5–20 min), peaking at 15 min. The recoveries decreased when the time was extended to 20 min, establishing 15 min as the optimal duration for subsequent experiments. Using the optimized MALLME conditions, an analytical method for the determination of 15 PYs in vegetable oils was developed in combination with the GC–MS/MS technique.

It should be noted that the DES-FFs material was collected following the extraction process, washed with methanol, and then dried. Subsequently, the S-Fe_3_O_4_ was reused in the preparation of DES-FFs material, which was then employed in MALLME experiments. The S-Fe_3_O_4_ material exhibited good reusability, with the capacity to undergo at least five successive cycles. The RSDs for the extraction recovery of 5 consecutive cycles was less than 10%.

### 3.4 Performance evaluation of analytical method

The analytical performance of the developed method was evaluated. To assess matrix effects, a series of standard solutions were added to the eluent after applying the DES-FFs-based MALLME procedure to cottonseed and olive oil samples. Standard solutions prepared in hexane and sample matrices were analyzed by GC–MS/MS, and calibration curves were established as shown in Table 3. Calibration curves in *n*-hexane and both sample matrices (cottonseed oil and olive oil) showed good linearity, with correlation coefficients exceeding 0.999. The slope ratios of solvent-to-matrix calibration curves were within 0.91–1.20, indicating effective sample purification by the MALLME method and significant suppression of the matrix effect (European Commission, 2021). Therefore, solvent-based calibration curves can be used for the quantitative analysis of real samples.

**Table 3:** Calibration curves of 15 PYs in solvent and matrix, LODs, and LOQs.

PYs	Linear range (ng/mL)	Linear equation (Solvent)	Linear equation (cottonseed oil matrix)	Slope ratio	Linear equation (olive oil matrix)	Slope ratio	LOD (ng/mL)	LOQ (ng/mL)
tefluthrin	10–1000	y = 5636.53× + 40.75 (R^2^ = 0.9993)	y = 6707.47×-3.20 (R^2^ = 0.9999)	1.19	y = 6456.09× + 0.46 (R^2^ = 0.9999)	1.15	2.35	7.82
bifenthrin		y = 17,290.88× + 108.47 (R^2^ = 0.9996)	y = 19,572.65×-0.68 (R^2^ = 0.9999)	1.13	y = 19,803.35× + 58.72 (R^2^ = 0.9997)	1.15	0.91	3.02
fenpropathrin		y = 385.58× + 29.33 (R^2^ = 0.9995)	y = 351.57×-0.20 (R^2^ = 0.9997)	0.91	y = 383.18× + 6.94 (R^2^ = 0.9998)	0.99	3.03	10.09
lambda-cyhalothrin		y = 2501.11× + 12.24 (R^2^ = 0.9993)	y = 2387.23× + 0.23 (R^2^ = 0.9998)	0.95	y = 2739.73× + 2.08 (R^2^ = 0.9999)	1.10	2.25	7.50
trans-permethrin		y = 1473.31× + 5.30 (R^2^ = 0.9998)	y = 1737.40× + 0.36 (R^2^ = 0.9997)	1.18	y = 1761.48×-4.37 (R^2^ = 0.9998)	1.20	2.94	9.81
cis-permethrin		y = 1493.40× + 15.93 (R^2^ = 0.9996)	y = 1769.00× + 0.68 (R^2^ = 0.9996)	1.18	y = 1693.61× + 10.27 (R^2^ = 0.9999)	1.13	2.42	8.06
cyfluthrin		y = 1186.43× + 9.89 (R^2^ = 0.9997)	y = 1280.12×-1.28 (R^2^ = 0.9998)	1.08	y = 1276.35× + 28.08 (R^2^ = 0.9996)	1.08	1.05	3.50
cypermethrin		y = 320.20× + 1.28 (R^2^ = 0.9995)	y = 357.32×-0.45 (R^2^ = 0.9998)	1.12	y = 336.14× + 2.12 (R^2^ = 0.9999)	1.05	2.09	6.95
flucythrinate		y = 1964.87× + 3.72 (R^2^ = 0.9997)	y = 2140.88×-2.77 (R^2^ = 0.9997)	1.09	y = 2069.80× + 1.83 (R^2^ = 0.9998)	1.05	2.08	6.92
beta-cypermethrin		y = 924.72× + 8.60 (R^2^ = 0.9997)	y = 1053.35×-1.14 (R^2^ = 0.9996)	1.14	y = 1052.69×-12.11 (R^2^ = 0.9993)	1.14	2.49	8.32
etofenprox		y = 13,053.40× + 84.19 (R^2^ = 0.9991)	y = 15,122.01× + 52.77 (R^2^ = 0.9991)	1.16	y = 14,592.80× + 87.47 (R^2^ = 0.9997)	1.12	1.20	3.98
fenvalerate		y = 568.69 x + 3.45 (R^2^ = 0.9994)	y = 608.68× + 0.95 (R^2^ = 0.9994)	1.07	y = 621.11× + 0.37 (R^2^ = 0.9995)	1.09	2.87	9.57
fluvalinate		y = 489.78×-0.43 (R^2^ = 0.9998)	y = 576.50× + 0.99 (R^2^ = 0.9999)	1.18	y = 484.40× + 2.55 (R^2^ = 0.9999)	0.99	2.19	7.29
esfenvalerate		y = 1300.91×-4.28 (R^2^ = 0.9999)	y = 1505.20×-0.15 (R^2^ = 0.9999)	1.16	y = 1260.54×-1.20 (R^2^ = 0.9999)	0.97	1.30	4.33
deltamethrin		y = 539.37×-5.55 (R^2^ = 0.9996)	y = 628.89× + 0.18 (R^2^ = 0.9996)	1.17	y = 550.45×-4.43 (R^2^ = 0.9998)	1.02	1.57	5.23

The sensitivity of the developed method was evaluated through ten replicate measurements following EPA SW-846 guidelines. As summarized in Table 3, the limits of detection (LODs) and quantification (LOQs) were defined as three and ten times the standard deviation (SD), respectively. LODs ranged from 0.91 to 3.03 ng/mL, and LOQs from 3.02 to 10.09 ng/mL for the 15 PYs. Method recovery was assessed via spiking experiments at three concentration levels. As presented in Table 4, recoveries ranged from 79.88% to107.90% for all PYs under optimized conditions. The relative standard deviations (RSDs) for intra-day precision (six replicates within 24 h) and inter-day precision (five replicates for consecutive days) were less than 5.06% and 6.92%, respectively. These results confirmed the reliability of the developed method for real-sample analysis.

**Table 4:** Recovery, intra-day and inter-day precisions of 15 PYs.

PYs	Spiked levels	Recovery (%)	Intra-day precision (*n* = 6)		Inter-day precision (*n* = 5)	
			The determined content (ng/mL)	RSD (%)	The determined content (ng/mL)	RSD (%)
tefluthrin	low	98.40–107.20	197.60 ± 2.31 (95%CI)^c^	1.46	197.90 ± 6.23 (95%CI)	3.59
	mid	96.25–101.00				
	high	86.32–93.44				
bifenthrin	low	99.60–104.80	200.10 ± 4.95 (95%CI)	3.09	199.89 ± 11.84 (95%CI)	6.76
	mid	97.85–102.40				
	high	89.74–95.32				
fenpropathrin	low	95.10–98.40	186.02 ± 4.03 (95%CI)	2.71	186.50 ± 6.05 (95%CI)	3.70
	mid	91.25–94.30				
	high	81.06–85.24				
lambda-cyhalothrin	low	98.80–103.80	195.30 ± 6.19 (95%CI)	3.96	196.70 ± 9.09 (95%CI)	5.27
	mid	94.50–100.60				
	high	90.18–92.22				
trans-permethrin	low	97.90–107.70	204.60 ± 5.06 (95%CI)	3.09	204.65 ± 10.99 (95%CI)	6.13
	mid	98.45–105.70				
	high	95.88–99.94				
cis-permethrin	low	99.40–105.30	202.10 ± 4.90 (95%CI)	3.03	201.60 ± 7.09 (95%CI)	4.01
	mid	98.15–103.50				
	high	93.94–96.98				
cyfluthrin	low	95.70–98.10	188.89 ± 6.08 (95%CI)	4.02	189.02 ± 9.26 (95%CI)	5.59
	mid	92.40–96.95				
	high	79.88–83.66				
cypermethrin	low	96.20–101.80	194.0 ± 3.97 (95%CI)	2.56	195.70 ± 7.62 (95%CI)	4.44
	mid	95.55–98.40				
	high	86.88–93.26				
flucythrinate	low	98.10–100.40	193.9 ± 4.38 (95%CI)	2.82	196.30 ± 6.59 (95%CI)	3.83
	mid	96.05–98.85				
	high	84.32–91.88				
beta-cypermethrin	low	100.70–107.90	189.9 ± 4.65 (95%CI)	3.06	190.20 ± 10.94 (95%CI)	6.56
	mid	98.90–100.10				
	high	91.70–94.82				
etofenprox	low	99.60–103.60	199.5 ± 6.56 (95%CI)	4.11	189.90 ± 10.67 (95%CI)	6.41
	mid	96.80–102.50				
	high	88.48–93.92				
fenvalerate	low	98.50–103.70	200.3 ± 7.61 (95%CI)	4.75	200.10 ± 9.19 (95%CI)	5.24
	mid	99.25–100.90				
	high	91.72–96.10				
fluvalinate	low	99.60–106.70	202.8 ± 8.21 (95%CI)	5.06	203.10 ± 12.32 (95%CI)	6.92
	mid	97.55–104.90				
	high	89.62–95.04				
esfenvalerate	low	97.90–104.50	197.56 ± 7.24 (95%CI)	4.58	198.12 ± 9.38 (95%CI)	5.40
	mid	95.55–101.80				
	high	87.44–93.06				
deltamethrin	low	98.40–103.30	202.7 ± 4.83 (95%CI)	2.98	201.90 ± 6.49 (95%CI)	3.67
	mid	99.45–103.00				
	high	92.12–94.98				

Note: ^c^: The determined content range within 95% confidence interval.

Low spiked concentration is 100 ng/mL; medium spiked concentration is 200 ng/mL; high spiked concentration is 500 ng/mL.

### 3.5 Sample analysis

The developed method was applied to analyze 15 PYs in cottonseed oil, sunflower oil, soybean oil, rapeseed oil, and olive oil under optimized conditions. Sample information for these refined and crude vegetable oils was provided in Table 5. To ensure reliability, triplicate tests were performed for each sample, analyzing both unspiked samples and samples spiked with 200 ng/mL PYs. As shown in Table 6, commercially available refined oils contained low levels of PYs residues, whereas crude oils showed high levels of PYs residues. Bifenthrin was detected in cottonseed oil (MM), soybean oil (JD1, JD3) and rapeseed oil (JC2). Deltamethrin was found in sunflower oil (JK1, JK3) and rapeseed oil (JC2). Cyfluthrin was detected in cottonseed oil (MM). The concentration of fenpropathrin in crude rapeseed oil (CM) exceeded China's MRL (3000 ng/mL) (Standardization Administration of China, 2021), which was 4612.57 ng/mL. The concentration of bifenthrin in refined rapeseed oil (JC2) exceeded China's MRL (100 ng/mL) (Standardization Administration of China, 2021), which was 195.40 ng/mL. Spiked sample recoveries ranged from 85.40% to 106.50%, demonstrating consistent performance of the developed method across various oil matrices. These results indicated that the method was effective in reducing matrix effect from diverse vegetable oils, particularly in impurity-rich crude oils, confirming the method reliability for accurate PYs quantification in vegetable oils. (See Table 6.)

**Table 5:** Vegetable oil samples information.

Vegetable oils	Samples	Sample codes
Cottonseed oil	Refined cottonseed oil 1	JM1
	Refined cottonseed oil 2	JM2
	Crude cottonseed oil	MM
Rapeseed oil	Refined rapeseed oil 1	JC1
	Refined rapeseed oil 2	JC2
	Crude rapeseed oil	CM
Sunflower oil	Refined sunflower oil 1	JK1
	Refined sunflower oil 2	JK2
	Refined sunflower oil 3	JK3
Soybean oil	Refined soybean oil 1	JD1
	Refined soybean oil 2	JD2
	Refined soybean oil 3	JD3
Olive oil	Olive oil 1	GL1
	Olive oil 2	GL2
	Olive oil 3	GL3

**Table 6:** Determined results of 15 PYs in vegetable oil samples.

PYs	Actual content vs. Spiked Recovery	Cottonseed oil			Sunflower oil		
		JM1	JM2	MM	JK1	JK2	JK3
tefluthrin	Content (ng/mL)	NF^d^	NF	NF	NF	NF	NF
	Recovery (%)	96.25–101.00			98.80–102.60		
bifenthrin	Content (ng/mL)	NF	NF	351.67 ± 5.08^e^	NF	NF	NF
	Recovery (%)	97.85–102.40			97.40–103.50		
fenpropathrin	Content (ng/mL)	NF	NF	NF	NF	NF	NF
	Recovery (%)	91.25–94.30			90.85–94.60		
lambda-cyhalothrin	Content (ng/mL)	NF	NF	NF	NF	NF	NF
	Recovery (%)	94.50–100.60			95.75–100.80		
trans-permethrin	Content (ng/mL)	NF	NF	NF	NF	NF	NF
	Recovery (%)	98.45–105.70			97.30–104.90		
cis-permethrin	Content (ng/mL)	NF	NF	NF	NF	NF	NF
	Recovery (%)	98.15–103.50			97.35–100.80		
cyfluthrin	Content (ng/mL)	NF	NF	812.37 ± 7.05	NF	NF	NF
	Recovery (%)	92.40–96.95			90.95–93.30		
cypermethrin	Content (ng/mL)	NF	NF	NF	NF	NF	NF
	Recovery (%)	95.55–98.40			91.90–96.25		
flucythrinate	Content (ng/mL)	NF	NF	NF	NF	NF	NF
	Recovery (%)	96.05–98.85			96.65–97.10		
beta-cypermethrin	Content (ng/mL)	NF	NF	NF	NF	NF	NF
	Recovery (%)	98.90–100.10			99.95–102.60		
etofenprox	Content (ng/mL)	NF	NF	NF	NF	NF	NF
	Recovery (%)	96.80–102.50			97.80–100.90		
fenvalerate	Content (ng/mL)	NF	NF	NF	NF	NF	NF
	Recovery (%)	99.25–100.90			94.05–96.30		
fluvalinate	Content (ng/mL)	NF	NF	NF	NF	NF	NF
	Recovery (%)	97.55–104.90			96.10–102.70		
esfenvalerate	Content (ng/mL)	NF	NF	NF	NF	NF	NF
	Recovery (%)	95.55–101.80			97.45–100.30		
deltamethrin	Content (ng/mL)	NF	NF	NF	19.73 ± 1.65	NF	26.60 ± 3.64
	Recovery (%)	99.45–103.00			93.05–98.40

**Table 6:** continued.

PYs	Actual content vs. Spiked Recovery	Soybean oil			Rapeseed oil			Olive oil		
		JD1	JD2	JD3	JC1	CM	JC2	GL1	GL2	GL3
tefluthrin	Content (ng/mL)	NF	NF	NF	NF	NF	NF	NF	NF	NF
	Recovery (%)	95.85–97.00			91.25–94.00			93.70–94.25		
bifenthrin	Content (ng/mL)	3.60 ± 1.06	NF	84.07 ± 2.48	NF	NF	195.40 ± 1.66	NF	NF	NF
	Recovery (%)	94.10–97.45			90.80–93.65			92.30–95.30		
fenpropathrin	Content (ng/mL)	NF	NF	NF	NF	4612.57 ± 11.57	NF	NF	NF	NF
	Recovery (%)	95.35–97.70			88.25–90.95			91.60–96.05		
lambda-cyhalothrin	Content (ng/mL)	NF	NF	NF	NF	NF	NF	NF	NF	NF
	Recovery (%)	93.70–97.75			91.90–92.35			99.20–101.80		
trans-permethrin	Content (ng/mL)	NF	NF	NF	NF	NF	NF	NF	NF	NF
	Recovery (%)	93.40–98.85			88.25–90.40			99.25–103.70		
cis-permethrin	Content (ng/mL)	NF	NF	NF	NF	NF	NF	NF	NF	NF
	Recovery (%)	89.15–93.10			91.80–92.35			98.05–102.50		
cyfluthrin	Content (ng/mL)	NF	NF	NF	NF	NF	NF	NF	NF	NF
	Recovery (%)	91.30–93.50			86.15–89.20			90.75–95.25		
cypermethrin	Content (ng/mL)	NF	NF	NF	NF	NF	NF	NF	NF	NF
	Recovery (%)	88.25–92.30			84.95–87.85			98.95–106.50		
flucythrinate	Content (ng/mL)	NF	NF	NF	NF	NF	NF	NF	NF	NF
	Recovery (%)	94.75–98.90			87.60–90.80			90.30–97.30		
beta-cypermethrin	Content (ng/mL)	NF	NF	NF	NF	NF	NF	NF	NF	NF
	Recovery (%)	93.90–95.90			88.10–92.75			95.75–100.30		
etofenprox	Content (ng/mL)	NF	NF	NF	NF	NF	NF	NF	NF	NF
	Recovery (%)	89.35–91.60			85.40–87.95			93.90–98.20		
fenvalerate	Content (ng/mL)	NF	NF	NF	NF	NF	NF	NF	NF	NF
	Recovery (%)	102.20–103.40			90.80–96.90			96.90–101.80		
fluvalinate	Content (ng/mL)	NF	NF	NF	NF	NF	NF	NF	NF	NF
	Recovery (%)	96.90–98.30			89.30–94.35			98.75–103.10		
esfenvalerate	Content (ng/mL)	NF	NF	NF	NF	NF	NF	NF	NF	NF
	Recovery (%)	93.10–96.20			88.00–91.05			96.30–98.55		
deltamethrin	Content (ng/mL)	NF	NF	NF	NF	NF	59.00 ± 4.28	NF	NF	NF
	Recovery (%)	102.10–105.90			89.40–92.60			98.25–104.80		

Note: ^d^: Not Found; ^e^: Mean ± SD.

The spiked concentration for the recovery experiment was 200 ng/mL; *n* = 3.

In Table 7, a comparison was conducted between the developed method and other methods for determining PYs in oil matrices as reported in the literature. These methods involved multi-step pretreatment processes, which were complex and time-consuming. For example, Esteve-Turrillas et al. (2005) first partitioned oil samples with acetonitrile-hexane (1:1), followed by elution through a combined column packed with basic alumina and C_18_. Sani et al. (2023) used α-terpineol:acetic acid DESs to extract several pyrethroid and carbamate pesticides from edible oils. After extraction, the DESs phase was separated using an ice-NaCl mixture bath and the collected DESs were decomposed with sodium carbonate solution. The solidified α-terpineol was then collected following an ice bath and diluted with acetonitrile for analysis. In contrast, a single-step MALLME based on DES-FFs developed in this study was more efficient and simpler. The DES-FFs was employed to extract PYs from oil samples, after which the extract was eluted with n-hexane for analysis. Meanwhile, the developed method demonstrated satisfactory recoveries (i.e. 79.88–107.90%) and comparable LODs (i.e. 0.91–3.03 ng/mL) of PYs in vegetable oil matrices relative to the literature values (i.e. recoveries: 62–112.79%; LODs: 0.01–160 ng/mL).

**Table 7:** Comparison results with other literatures.

References	Solvents/Absorbents	Methods	Oils	PYs	LODs (ng/mL)	Recovery (%)
M.Z. Sani et al. (2023)	DESs	DLPME-HPLC-DAD	Sunflower oil, corn oil, olive oil, rapeseed oil, peanut oil, sesame oil	Cypermethrin, deltamethrin, cypermethrin, cyfluthrin	0.2–0.8	84–91
P. Deme et al. (2013)	Acetonitrile	LLME-GC-NCI-MS/MS	Sunflower oil, rice bran oil, peanut oil	Deltamethrin, cyfluthrin, cypermethrin	0.02–0.1	58–82
F.A. Esteve-Turrillas et al. (2005)	Acetonitrile-Hexane	SPE-GC–MS/MS	Olive oil, sunflower oil, corn oil and soybean oil	Tetramethrin, Bifenthrin, Phenothrin, Cyhalothrin, Permethrin, Cyfluthrin, Cypermethrin, Flucythrinate, Esfenvalerate, Fluvalinate and Deltamethrin	1.0–5.0	91–104
M.A. Farajzadeh et al. (2014)	1,1,2-trichloroethane	DLLME-GC-FID	Corn oil, colza oil, olive oil and sunflower oil	Deltamethrin, cyhalothrin, permethrin, fenpropathrin	20–160	85.5–109.3
X. Yu et al. (2017)	PSt/MNPs	MSPE-HPLC	Soybean oil, canola oil, sunflower oil, corn oil and virgin olive oil	Tetramethrin, deltamethrin, cyfluthrin, cypermethrin permethrin, decamethrin, fenvalerate, acrinathrin	0.0290–0.0658	83.18–112.79
P. Deme et al. (2014)	Acetonitrile	DSPE-GC-NCI-MS/MS	Sunflower oil, rice bran oil, ground nut oil	Allethrin, cyfluthrin, cypermethrin, flumethrin, deltamethrin	0.01–1	62–110
This study	DES-FFs	VALPME- GC–MS/MS	Cottonseed oil, sunflower oil, soybean oil, rapeseed oil, olive oil	Tefluthrin, Bioresmethrin, Bifenthrin, Fenpropathrin, Lambda-cyhalothrin, Trans-permethrin, Cis-permethrin, Cyfluthrin, Cypermethrin, Beta-cypermethrin, Flucythrinate, Etofenprox, Fluvalinate, Fenvalerate, Esfenvalerate, Deltamethrin	0.91–3.03	79.88–107.90

Although a simple pretreatment was adopted in this study, a comparison of the slopes of calibration curves in solvent and matrices indicated effective sample purification and significant suppression of the matrix effect. In the MALLME system, it was likely that the DESs achieved high extraction efficiency of PYs through π-π stacking interactions between sesamol/COU and the target compounds. Moreover, due to the hydrophilicity-polarity matching among DESs, oil and the elution solution, DESs could not dissolve in the oil and n-hexane, while n-hexane efficiently extracted PYs from the DESs phase. These mechanisms collectively reduced the matrix effect from the oil samples, confirming that the developed method was suitable for the multi-residues analysis of PYs in vegetable oils.

## 4 Conclusion

In this study, a ChCl/sesamol/COU-based DES-FF was prepared and used in the MALLME and determination of 15 PYs in vegetable oil with GC–MS/MS method. The DES-FFs with small-sized Fe_3_O_4_ nanoparticles demonstrated enhanced stability and facilitated rapid phase separation in less than 10 s. And ChCl/sesamol/COU-based DESs may exhibit stronger π-π stacking interactions with PYs, and have suitable hydrophilicity and polarity to extract PYs from vegetable oil matrices. Material characterization techniques (e.g., XRD, HTEM, FT-IR, DSC, TGA, and Hysteresis loops, etc.) revealed the successful synthesis of DES-FFs materials and their suitability for use as magnetic adsorbents in MALLME. The method yielded satisfactory calibration curves, with correlation coefficients exceeding 0.999, and matrix effect (e.g. from cottonseed oil to olive oil) were significantly suppressed after purification with the MALLME technique. The LODs and LOQs for the 15 PYs ranged from 0.91 to 3.03 ng/mL and 3.02 to 10.09 ng/mL, respectively. Recoveries for all PYs ranged from 79.88% to 107.90%, with good precision (RSDs ≤6.92%). The practical application of the developed method was verified using real refined and crude vegetable oil samples. Additionally, the developed method was more efficient and simpler than those reported in the literature, providing a simple, single-step pretreatment platform for fats and oils samples.

## CRediT authorship contribution statement

**Jingjing Yu:** Writing – original draft, Supervision, Funding acquisition. **Yuxin Liu:** Methodology, Investigation. **Cong Wang:** Software, Investigation. **Mantang Chen:** Funding acquisition, Data curation. **Cong Nie:** Supervision, Project administration. **Wei Liu:** Writing – review & editing, Supervision.

## Declaration of competing interest

The authors declare that they have no known competing financial interests or personal relationships that could have appeared to influence the work reported in this paper.

## Data Availability

Data will be made available on request.

## Appendix A Supplementary Data

Supplementary material Image 1
