# Supplementary material for: Deep eutectic solvent-Ferrofluid based single-step magnetic-assisted liquid-liquid microextraction for Pyrethroid residues determination in vegetable oils by GC–MS/MS
Source: Food Chem X. 2026 Jan 24;34:103576. doi: 10.1016/j.fochx.2026.103576 (PMC12865580; doi:10.1016/j.fochx.2026.103576)
Supplement: Supplementary file 1 — Supplementary material [file mmc1.docx]

**Supplement information**

**Deep Eutectic Solvent-Ferrofluid Based Single-Step Magnetic-assisted Liquid-liquid Microextraction for Pyrethroid Residues Determination in Vegetable Oils by GC-MS/MS**

Jingjing Yu^1^*, Yuxin Liu^1,2^, Cong Wang^1^, Mantang Chen^1^, Cong Nie^1^, Wei Liu^2^*

^1^Zhengzhou Tobacco Research Institute of CNTC, Zhengzhou 450001, P. R. China.

^2^College of Food Science and Technology, Henan University of Technology, Lianhua Street, Zhengzhou 450001, P. R. China.

*Corresponding author. Tel/Fax: 086-18623717860

E-mail address: yujingjing307@163.com (J. Yu), [liuwei307@hotmail.com (W](mailto:liuwei307@hotmail.com%20(W). Liu)

- 1. **XRD characterization of small-sized Fe_3_O_4_ nanoparticles (S-Fe_3_O_4_).**

The XRD spectra of small-sized Fe_3_O_4_ nanoparticles (S-Fe_3_O_4_) is shown in Figure S1. Six peaks observed at 2θ values of 30.28° (200), 35.48° (311), 43.20° (400), 53.44° (422), 57.06° (511), and 62.80° (440) is consistent with the crystallographic planes of the characteristic diffraction peaks of Fe_3_O_4_ nanoparticles, indicating that S- Fe_3_O_4_ was successfully synthesized.

**1.2 Thermogravimetric analysis (TGA) curve of DESs (ChCl/Sesamol/COU, 1:3:1)**

DESs prepared from ChCl/Sesamol/COU (1:3:1) were characterized using thermogravimetric analysis (TGA). As shown in Figure S2, the TGA curve was divided into three parts: the maximum mass loss (about 65.07%) was observed at 163.58 ºC, which corresponds to the decomposition of sesamol. And a smaller mass loss (about 12.04%) was observed at 200.84 ºC, which corresponds to the sublimation of coumarin (COU). Then a large mass loss (about 22.89%) was observed at 300.14 ºC, corresponding to the decomposition of ChCl. Compared with that of sesamol in single-component state (214 ºC), and the decomposition temperature of sesamol in DESs decreased to a certain extent (163.58 º C). But the sublimation temperature of coumarin (COU) in DESs (177.05 ºC) is basically close to that of coumarin in single-component state (175 °C). And the decomposition of ChCl in DESs decreased to 233.48 ºC, compared with that of ChCl in single-component state (about 300 ºC). These phenomena are consistent with the conclusion that “the thermal stability of DESs is not higher than that of its component monomers” as mentioned in the literature (Farajzadeh et al., 2014).

**1.3 Differential Scanning Calorimetry（DSC）curve of DESs (ChCl/Sesamol/COU, 1:3:1)**

DSC curve (Figure S3) of DESs (ChCl/Sesamol/COU, 1:3:1) exhibits the glass transition temperature (Tg) at about -67.06 ^o^C, which is significantly lower than the melting point of its individual constituents, ChCl, Sesamol, and COU (melting point of ChCl, Sesamol and COU being 302 ^o^C, 62-65 ^o^C and 68-70 ^o^C respectively). In addition, Tg obtained from heating curve is -67.06 ^o^C, while cooling curve gives a similar Tg (i.e. -65.87 ^o^C). It indicates that the ratio of 1:3:1 is suitable for the formation of DESs and there are no unreacted monomers. These results confirmed the formation of DESs.

**Captions**

Figure S1 XRD pattern of small-sized Fe_3_O_4_ nanoparticles (S- Fe_3_O_4_).

Figure S2 TGA curve of DESs (ChCl/Sesamol/COU, 1:3:1).

Figure S3 DSC curve of DESs (ChCl/Sesamol/COU, 1:3:1).

Figure S1 XRD pattern of small sized Fe_3_O_4_ nanoparticles (S-Fe_3_O_4_).


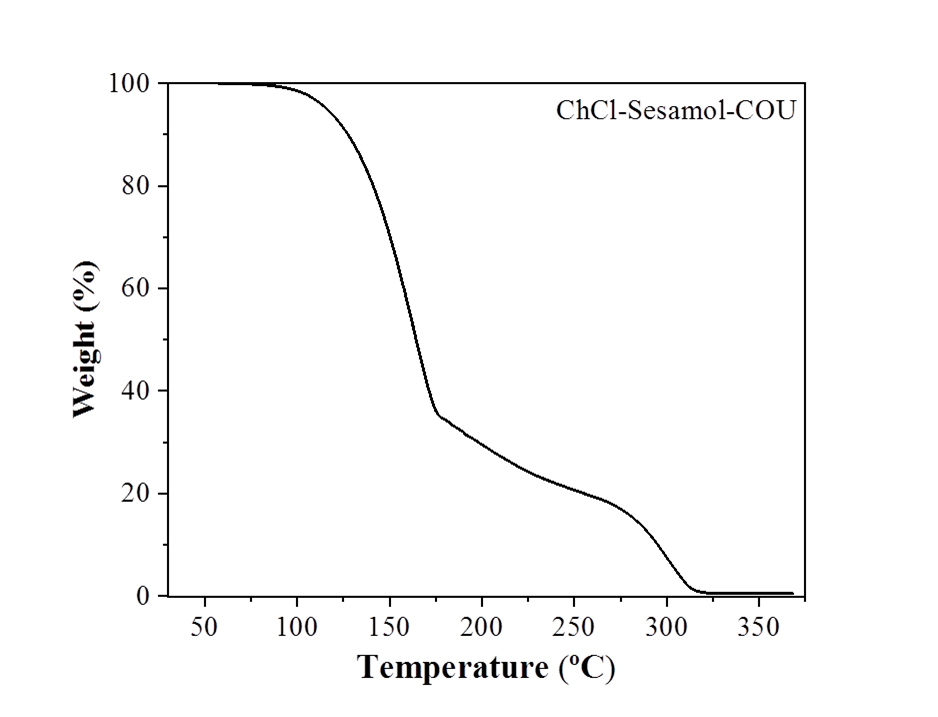


Figure S2 TGA curve of DESs (ChCl/Sesamol/COU, 1:3:1).


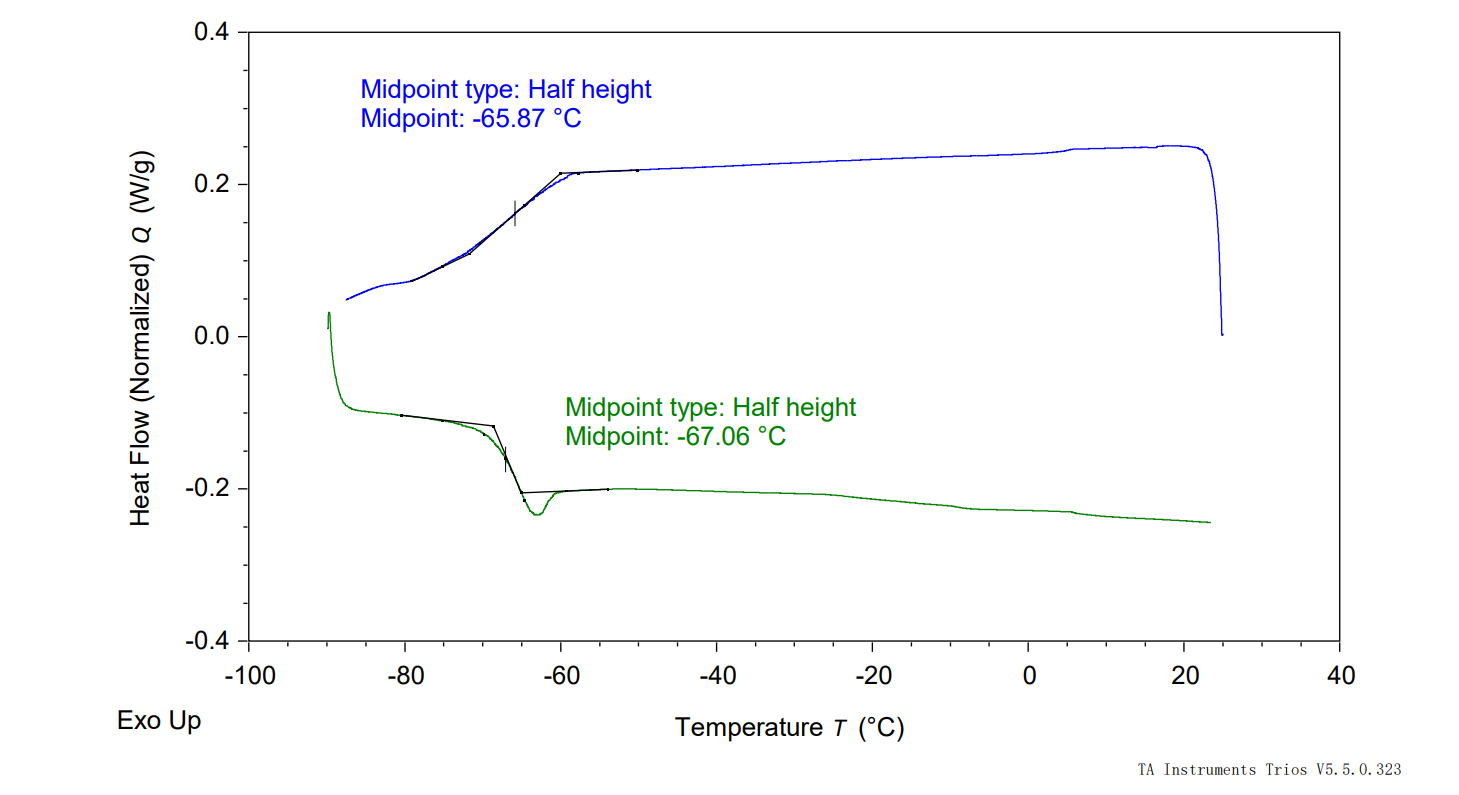


Heatting

cooling

Figure S3 DSC curve of DESs (ChCl/Sesamol/COU, 1:3:1).
